# Integrated Epithelial Models Reveal Anti-Inflammatory and Barrier Modulatory Properties of Ozoile in Inflammatory Bowel Disease

**DOI:** 10.3390/antiox15060664

**Published:** 2026-05-25

**Authors:** Daniele Bravoco, Giuseppina di Paola, Valeria Lucci, Carlo Calabrese, Serena Vella, Domenico Montesano, Rosarita Tatè, Rebecca Leandri, Gionata De Vico, Salvatore Valiante, Teresa Barra, Geppino Falco, Giuliana Napolitano, Pellegrino Mazzone

**Affiliations:** 1 Istituto di Ricerche Genetiche ‘Gaetano Salvatore’, Biogem Scarl, 83031 Ariano Irpino, Italy; 2 Department of Public Health, Federico II University of Naples, 80131 Naples, Italy; 3 CaWUR srl, 83031 Ariano Irpino, Italy; 4 Department of Biology, University of Naples Federico II, 80147 Naples, Italy; rebecca.leandri@unina.it (R.L.); gionata.devico@unina.it (G.D.V.);; 5 Istituto di Ricovero e Cura a Carattere Scientifico, IRCCS-CROB, Referral Cancer Center of Basilicata, Rionero in Vulture, 85028 Potenza, Italy; 6 Erbagil s.r.l., Via L. Settembrini, 13, 82037 Telese Terme, Italy; s.vella@erbagil.com (S.V.);; 7 Institute of Genetics and Biophysics ‘A. Buzzati-Traverso’, National Research Council, Via Pietro Castellino, 111, 80131 Naples, Italy; 8 National Institute of Biostructure and Biosystems (INBB), Via dei Carpegna 19, 00165 Rome, Italy; 9 Institute of Polymers, Composites and Biomaterials, National Research Council (IPCB-CNR), Viale Kennedy, 54, Mostra d’Oltremare—Pad. 20, 80078 Pozzuoli, Italy

**Keywords:** inflammation, organoids, preclinical models, natural compounds

## Abstract

*Background*: Inflammatory bowel disease (IBD) is a chronic inflammatory condition, with therapy-resistant patients undergoing surgery and a high risk of developing colorectal cancer. Novel therapeutic approaches have shown limited efficacy in IBD treatment, highlighting the need for safer and more personalized strategies. The potential of natural compounds to modulate inflammation suggests their use as a potential adjunct therapy for IBD patients. *Methods*: Intestinal epithelial cells organoids (IECOs) were derived from IBD and non-IBD tissues from IBD patients, and levels of inflammation markers and epithelial barrier permeability were assayed using qRT-PCR, WB, IF and leaking assays in the presence of Ozoile, an extra virgin olive oil enriched in ozonides. The Luciferase-based IBD-like organoid platform was generated for preliminary screening of anti-inflammatory drugs. *Results*: In this study, we showed that IBD-ECOs recapitulate tissue architecture and pathological state. We showed that Ozoile has anti-inflammatory and epithelial barrier modulatory effects and that the Luciferase IBD-like organoid model is sensitive to anti-inflammatory compounds. *Conclusions*: Using IECOs, the specific anti-inflammatory and regenerative properties of Ozoile were assessed. Notably, our study highlights the potential of an IBD-like organoid platform to use in high-throughput screenings for rapid selection of anti-inflammatory drugs.

## 1. Introduction

Inflammatory bowel disease (IBD) is a group of immune-mediated disorders characterized by chronic inflammation of the gastrointestinal tract, mainly including Crohn’s disease (CD) and ulcerative colitis (UC) [1]. IBD is a progressive and disabling condition characterized by heterogeneous clinical phenotypes that significantly impair patients’ quality of life. Only a subset of patients achieves sustained long-term clinical remission, while many experience chronic, relapsing disease courses that require ongoing treatment and monitoring [2,3]. Over the past decades, the global incidence of IBD has been constantly increasing, with a high prevalence in Western countries and a significant expansion also in developing regions [4,5]. Paralleling the constant increase in affected people, European and American guidelines have historically recommended a stepwise therapeutic approach to manage IBD, involving anti-inflammatory drugs and, more recently, biological agents. However, recent studies suggest that early and more intensive therapy with certain medications may lead to better clinical outcomes than traditional incremental strategies (e.g., early biologic therapy has been associated with improved remission and reduced complications compared with late or step-up treatment) [6,7]. However, the low success rate of conventional drugs, their significant side effects and the development of drug resistance, combined with the high costs of biologics-based therapies, represent a significant economic burden on healthcare systems, considering the small percentage of patients achieving sustained clinical remission [8,9]. These shortcomings have prompted research into safer natural compounds, especially plant-derived natural products, with anti-inflammatory and antioxidant properties. Natural compounds thus represent promising complementary strategies for patients with IBD to avoid the side effects of combining standard medical treatments [10,11]. Notably, due to the multifaceted nature of IBD, experimental models mimicking the in vivo complexity of chronic pathology are difficult to obtain. Recently, patient-intestinal organoids, the three-dimensional structures derived from epithelial stem cells isolated from intestinal biopsies, have gained recognition as a robust pre-clinical model in IBD as they accurately recapitulate the histological architecture, molecular characteristics and functional properties of the tissue of origin, thus providing consistent advantages compared to immortalized tumor cells, animal models or primary biopsies. Although patient-derived organoids are increasingly recognized as powerful preclinical models for modeling human diseases and for designing personalized therapeutic strategies, their use in drug-screening pipelines remains limited. In this study, to contribute to an advancement in this field, we employed intestinal patient-derived organoids to establish a luciferase-based IBD-like model platform to evaluate its potential for anti-inflammatory drug screening pipelines. We also analyzed anti-inflammatory and epithelial barrier healing activity of a novel natural compound, Ozoile, a patented formulation of stable ozonides combined with extra-virgin olive oil, chemically characterized in Vella et al. (2025) [12]. Our results highlight the potential of natural compounds and especially Ozoile as an adjunct therapy for inflammatory bowel disease. By integrating organoid models with the study of a natural bioactive compound, this work lays out the groundwork for the use of natural therapeutic supplements in IBD and for the development of innovative and safer strategies for IBD management.

## 2. Materials and Methods

### 2.1. Isolation and Culture of Intestinal Epithelial Cell Organoids

Biological samples were obtained from non-inflamed and inflamed tissues of patients with UC or CD enrolled at Sant’Orsola Hospital (Italy) who gave informed consent (Table S1). The study was conducted in accordance with the Declaration of Helsinki, and approved by the Comitato Etico Campania Nord, and Azienda Ospedaliera “San Giuseppe Moscati”, Avellino, Prot. CECN 2070 on 22 March 2023. Informed consent was obtained from all subjects involved in the study. The fresh biopsies were collected in the tissue storage medium (Miltenyi, Bologna, Italy) and then digested for 1 h at 37 degrees with Collagenase and Hyaluronidase. Subsequently, cells were filtered using a 70 μm strainer in the 15 mL conical tube, centrifuged for 5 min at 300× *g* and resuspended in 50 μL droplets of Matrigel (Corning, Turin, Italy). Finally, the cells were maintained in Intestinal organoids medium (DMEM/F12 supplemented with 10 mM HEPES, 1% Pen/Strep, 2 mM Glutamax, 1x B27, 1x N2, 1 mM N-Acetylcystine, 50 ng/mL EGF, 10 mM Nicotinamide, 50% (*v*/*v*) Wnt/R-Spondin/Noggin (l-WRN) Conditioned Medium, 10 nM Gastrin, 2 μM A83-01, 10 μM SB202190, 10 μM Y-27632, 100 μg/mL Primocin) at 37 °C in 5% CO_2_, as previously described by Sato et al. (2011) [13,14,15]. The culture medium was replaced with a fresh medium every two days.

### 2.2. Cell Culture

Caco-2 (HTB-37™) and HEK293T (CRL-3216™) cells were purchased from ATCC (Manassas, VA, USA) and were cultured in Dulbecco’s modified Eagle’s medium supplemented with 10% FBS (Gibco, Waltham, MA, USA) and 1% Pen/Strep (Gibco, Waltham, MA, USA) at 37 °C in 5% CO_2_.

### 2.3. RNA Extraction

Total RNA was isolated from organoids and tissues by PureLink™ RNA Mini Kit (Invitrogen, Waltham, MA, USA) according to the manufacturer’s instructions. The quantity and quality of RNA were measured using the NanoDrop 1000 Spectrophotometer (Thermo Fisher Scientific, Waltham, MA, USA).

### 2.4. qPCR Analysis

For qPCR analysis, 1 μg of total RNA was reverse transcribed using the Quantitec reverse transcription kit (Qiagen, Hilden, Germany) according to the manufacturer’s recommendations. qPCR analyses were performed using 10 ng cDNA per well in triplicate with the SYBR green master mix (Applied Biosystems, Waltham, MA, USA). All experiments were performed on a real-time PCR system (Applied Biosystems) and a QuantStudio 7 Flex system (Applied Biosystems, Waltham, MA, USA). Fold induction was calculated using the ΔΔCt method [16,17,18], while the normalization was performed using the mean of two housekeeping genes: Beta-2-Microglobulin (B2M) and Actin. The gene-specific primers are reported in Table S2.

### 2.5. Treatments

Cells and organoids were treated with TNFα (Miltenyi Biotec, Bergisch Gladbach, Germany), Dexamethasone (Sigma-Aldrich, St Louis, MO, USA) and Capsaicin (Selleckchem, Houston, TX, USA) at the concentrations of 20 ng/mL, 10 μM and 50 μM, respectively. Dexamethasone and TNFα were dissolved in DMEM high glucose or IECOs Medium, while Capsaicin was dissolved in dimethyl sulfoxide (DMSO; Sigma-Aldrich, St Louis, MO, USA). Ozoile^®^ Food, extra virgin olive oil rich in ozonides, is obtained through a green technology, according to a patented process (EP3900821) by reaction of a specific amount of ozone with the olefin bonds of the fatty acids of the organic extra virgin olive oil +Oil^®^ obtained from native olives grown organically in Sannio (Benevento, Italy). The details on the isolation, identification, and full structural characterization of stable ozonides of Ozoile were reported in Vella et al., 2025 [12]. Ozoile was diluted in isopropanol, used at a concentration of 2 mg/mL and dissolved in DMEM high glucose or IECOs Medium. The treatments include the +Oil^®^, the extra virgin oil that is not ozonized, which is also diluted in isopropanol and used as a negative control. IECOs and Caco-2 cells were treated with or without TNFα for 4 h, 10 days post-seeding for IECOs and 1 day post-seeding for Caco-2, respectively, and then drugs were added and left for 24 h more. Culture media and solvent controls were included and used for calculating treatment efficacy relative to the control.

### 2.6. Plasmids

To generate the phageNF-κBLucPuro construct, the puromycin sequence was amplified by PCR from the pLentiCMVLucPuro basic vector (Addgene, Watertown, MA, USA, #17477) and inserted into the NdeI/ClaI (New England Biolabs, Ipswich, MA, USA) sites of the phageNF-κB-TA-Luc-UBC-GFP vector (Addgene #49343). The construct was verified by restriction analysis and sequencing.

### 2.7. Lentivirus

To produce lentiviruses encoding NF-κB inducible luciferase, HEK-293T cells were co-transfected with phageNF-κBLucPuro and pMD2.G and psPAX2 packaging plasmids (Addgene#12259; Addgene #12260) using the calcium phosphate method [19]. For the establishment of stable cell lines, HEK-293T medium containing lentiviral particles was filtered through a 0.45 μm filter and used immediately, combined with 0,01 mg/mL polybrene to inoculate Caco2 cells. After one day, the resulting pNF-κBLucPuroCaco2 cells were selected with 5 µg/mL puromycin for five days. To generate stable organoid lines, lentiviral particles were filtered through a 0.45 μm filter and concentrated by centrifugation at 20,000 rpm for 4 h at 4 °C. Then IECOs were cellularized, and cells were resuspended with lentiviral particles containing 0.01 mg/mL polybrene and centrifuged at 600× *g* for 1 h. Therefore, the supernatant was discarded, and the precipitated cells were resuspended in Matrigel and seeded in a 24-well plate and incubated at 37 °C. After 15 min, culture medium was added to the drops. Seven days after transduction, 1 µg/mL puromycin was added to select for stably transduced pNFκBLucIECOs.

### 2.8. Luciferase Assay

To assess NF-κB-dependent promoter activation upon drug treatment, luciferase reporter assays were performed. Briefly, following treatments, Caco-2 cells and intestinal epithelial organoids (IECOs) were lysed using the Luciferase Assay System (Promega, Madison, WI, USA) according to the manufacturer’s instructions. After lysis, samples were clarified by centrifugation to remove cellular debris, and equal amounts of supernatant were transferred into white opaque 96-well plates suitable for luminescence detection. Luciferase activity was measured by adding the appropriate luciferase substrate reagent, and luminescence was immediately recorded using a Synergy HTX multimode microplate reader (Agilent BioTek, Santa Clara, CA, USA), with consistent acquisition settings applied across all experimental conditions.

### 2.9. MTT Assay

Caco-2 cells (1 × 10^3^ cells/well) were plated in 96-well plates. After Ozoile treatment, cell viability was assessed by adding MTT 0.5 mg/mL for 3 h at 37 °C, then the medium was removed and replaced with 100 μL of dimethyl sulfoxide (DMSO) at 37 °C for 1 h; otherwise, IECOs were plated in a 96-well plate. Organoids’ viability was evaluated by adding 0.5 mg/mL MTT for 2 h at 37 °C, then the medium was replaced with 2% SDS to solubilize Matrigel, for 2 h at 37 °C. Subsequently, SDS was replaced with 100 μL of DMSO at 37 °C for 1 h. The absorbance values of the solution in each well, for both cells and organoids, were detected at 570 nm using an Envision XCITE Multimode Plate Reader Perkin Elmer Waltham, MA, USA). All MTT experiments were performed in triplicate. Cell and organoid viability were expressed as the percentage of absorbance values in treated samples compared to the control.

### 2.10. Immunofluorescence

Intestinal organoids were recovered by removing the medium and adding Corning Cell Recovery medium to depolymerize Matrigel. Organoids were allowed to settle under gravity for 5 min and then washed three times with 1× PBS. Subsequently, organoids were fixed for 1 h at RT with 4% paraformaldehyde and washed three times with 1× PBS. Then, organoids were permeabilized for 10 min with 0.1% Triton X-100 in PBS, followed by a blocking solution (5% BSA, 0.2% Triton X-100 in PBS) at RT for 2 h. Organoids were then incubated overnight at 4 °C with Mouse Monoclonal MUC2 primary antibody (1:200; Sc-515032; Santa Cruz, TX, USA), Rabbit Monoclonal Villin primary antibody (1:200; Ab130751; Abcam, Cambridge, UK), and Rabbit Monoclonal Lysozyme primary antibody (1:200; Ab108508; Abcam). After three washes with 1× PBS, cells were incubated with the Alexa Fluor 488-Goat Anti-Rabbit IgG secondary antibody (1:1000; A-11008; ThermoFisher Scientific, Waltham, MA, USA) for 1 h at RT. Organoids were carefully resuspended in 50 µL of 1× PBS and transferred to a poly-L-lysine-coated 8-well chamber slide, where they were allowed to settle for 30 min. PBS was removed, and the slide was mounted using ProLong™ Glass Antifade Mountant with NucBlue™ (P36981; ThermoFisher Scientific, Waltham, MA, USA). Imaging was performed using an Olympus FLUOVIEW FV3000 confocal microscope with a ×40 objective. For Caco-2 immunofluorescence staining, cells were seeded in a 24-well plate with a coverslip. Upon treatment with compounds, cells were fixed with 4% paraformaldehyde and washed three times with 1× PBS. Cells were permeabilized for 10 min with 0.1% Triton X-100 in PBS, followed by a blocking solution (5% BSA, 0.2% Triton X-100 in PBS) at RT for 1 h. Cells were then incubated overnight at 4 °C with Rabbit Monoclonal NF-κB primary antibody (8242S Cell Signaling Technology, Danvers, MA, USA), anti-INOS (RM1017, ab283655), and anti-ZO-1 (D7D12, #8193), anti γH2AX (phospho S139, ab81299). After three washes with 1× PBS, cells were incubated with the appropriate Alexa Fluor 488 and Alexa Fluor 594 secondary antibody (1:1000, ThermoFisher Scientific) for 1 h at RT and then washed again with 1× PBS. DAPI was used to stain nuclei [20,21]. Coverslips were mounted on the microscopy glasses and visualized at an Olympus FLUOVIEW FV3000 confocal microscope (Tokio, Japan).

### 2.11. Western Blot Analysis

Total protein was extracted with RIPA buffer using the following formulation: 100 mM Tris pH7.4, 100 mM NaCl, 2 mM Na3VO4, 0.5% deoxycholate, 2% NP40, 1 mM EDTA, 1 mM NaF, 1 mM PMSF, 20 mM Na4P2O7, and 1× Protease Inhibitor Cocktail (Sigma) [22,23]. Protein concentrations were determined using the Bio-Rad protein assay kit according to the manufacturer’s instructions. Fifty micrograms of protein lysate were run on SDS-PAGE and transferred onto the nitrocellulose membrane. The membranes were incubated with anti-P-NF-κB (93H1, #3033S Cell Signaling Technology), anti-NF-κB (D14E12, # 8242 Cell Signaling Technology) and anti-β-ACTIN (A2228, Sigma Aldrich), followed by incubation with HRP-conjugated anti-mouse IgG or anti-rabbit IgG (1:2500; Amersham Biosciences, Buckinghamshire, UK). Blots were developed using the ECL system (Santa Cruz, Dallas, TX, USA).

### 2.12. ELISA Assay

IL-8 levels were measured in the supernatant of both non-IBD and IBD organoids using the Elabscience (Wuhan, China) Human IL-8 ELISA kit (cat. E-EL-H6008) as reported in the manufacturer’s instructions. Briefly, samples were diluted with the provided sample diluent in a 1:4 ratio. An eight-point calibration curve was prepared using a freshly suspended calibrator, and standard serial dilutions were prepared ranging from 0 to 500 pg/mL. In a final volume of 50 µL, the calibration curve standards and samples were pipetted on a Q-Plex^TM^ Array 96-well plate. The plate was sealed, incubated for 90 min, then washed with the provided wash solution, and the detection mix antibody solution was added. After 60 min incubation at 37 °C, the plate was washed; then, Streptavidin-HRP was added and incubated for another 30 min. After five final washes, the freshly prepared substrate solution was added, and the plate was read immediately at 450 nm using the Perkin Elmer EnSpire 2300 MultiMode Microplate Reader, Waltham, MA, USA. All samples and standards were measured in triplicate. All incubations were performed at room temperature (25 °C) on a shaker set to 500 rpm.

### 2.13. DCFDA Assay

Antioxidant activity was evaluated using 2′,7′-dichlorofluorescein diacetate (DCFDA). Briefly, 3 × 10^3^ Caco-2 cells were treated with 2 μg/μL of the natural compounds either before or during exposure to 1 mM (3%) H_2_O_2_ for 2 h. After treatment, the cells were washed with PBS and incubated in a fresh medium containing DCFDA (30 μM) for 45 min. This incubation time was selected based on the manufacturer’s recommendations and commonly used protocols, ensuring stable and reproducible detection of intracellular ROS levels. DCFDA was then removed by washing with PBS 1×, and the cells were harvested. ROS levels were measured using a Synergy H1 microplate reader (Agilent BioTek, Santa Clara, CA, USA). Fluorescence intensity from DCFDA-treated cells was compared with that of untreated control cells. Trolox, a water-soluble analog of vitamin E, was used as a positive control due to its antioxidant activity. Data are presented as mean ± SD of four determinations. The mean and standard deviation were calculated from biological triplicates using GraphPad Prism 10 software (GraphPad, San Diego, CA, USA).

### 2.14. Caco-2 Cells Differentiation into Enterocytes and Treatments

For intestinal barrier formation in vitro, Caco-2 cells were differentiated into enterocytes. Briefly, Caco-2 cells were seeded at confluence and grown for 14 days in Dulbecco’s modified Eagle’s medium supplemented with 10% FBS (Gibco) and 1% Pen/Strep (Gibco) at 37 °C in 5% CO_2_. Medium was replaced every 2 days, and differentiation was assessed by the formation of tall and regular microvilli and by the presence of properly formed Tight Junctions (TJs) (see Figure S2).

### 2.15. Intestinal Epithelial Barrier Permeability Assay

For the paracellular permeability assay, Caco-2 cells were seeded at confluence on a permeable PET membrane (0.45 μm diameter) in the Live Box 2 bioreactor systems purchased from IVTech Srl (IVTech Srl., Massarosa, LU, Italy). Paracellular permeability was evaluated by measuring the diffusion of Lucifer Yellow (#L0144 Sigma-Aldrich) that was added apically. Withdrawals from apical and basolateral compartments were done after 30, 60, 90, 120 and 240 min. Fluorescence was measured by a spectrofluorometer (Synergy HTX, Agilent Biotek, Santa Clara, CA, USA) according to the manufacturer’s instructions, and permeability was calculated as the percentage of Lucifer Yellow that passed from the apical to the basolateral chamber. Data are presented as mean ± SD of three determinations. The mean and standard deviation were calculated from biological duplicates using GraphPad Prism 10 software (GraphPad, San Diego, CA, USA).

### 2.16. Ultrathin Slides Preparation for Transmission Electron Microscopy Analyses

Caco-2 cells were fixed in 2.5% glutaraldehyde in cacodylate buffer for 1 h. Then, they were rinsed in the same buffer and postfixed in 1% osmium tetroxide for 1 h at room temperature, dehydrated in graded alcohol, and embedded in Epon 812 (PolyScience, Warrington, PA, USA). Semi-thin sections (200 nm) were cut on an ultracut UCT ultramicrotome (Leica Microsystems, Wetzlar, Germany), stained with 1% toluidine blue in water solution and examined by light microscopy. Ultrathin sections (60–70 nm), obtained with the same ultramicrotome from chosen areas, were collected onto 300-mesh grids and contrasted with uranyl acetate. Grids were examined using a FEI Tecnai G^2^ S-TWIN 200 kV transmission electron microscope (TEM) (FEI Company, Dawson Creek Drive, Hillsboro, OR, USA) operating at 120 kV with a LaB6 (Lanthanum hexaboride) source; micrographs were taken with TEM User Interface Software (TUI Software Version 4.4.1)

## 3. Results

### 3.1. Patient-Derived Intestinal Organoids Recapitulate the Tissue of Origin

**Figure 1:**
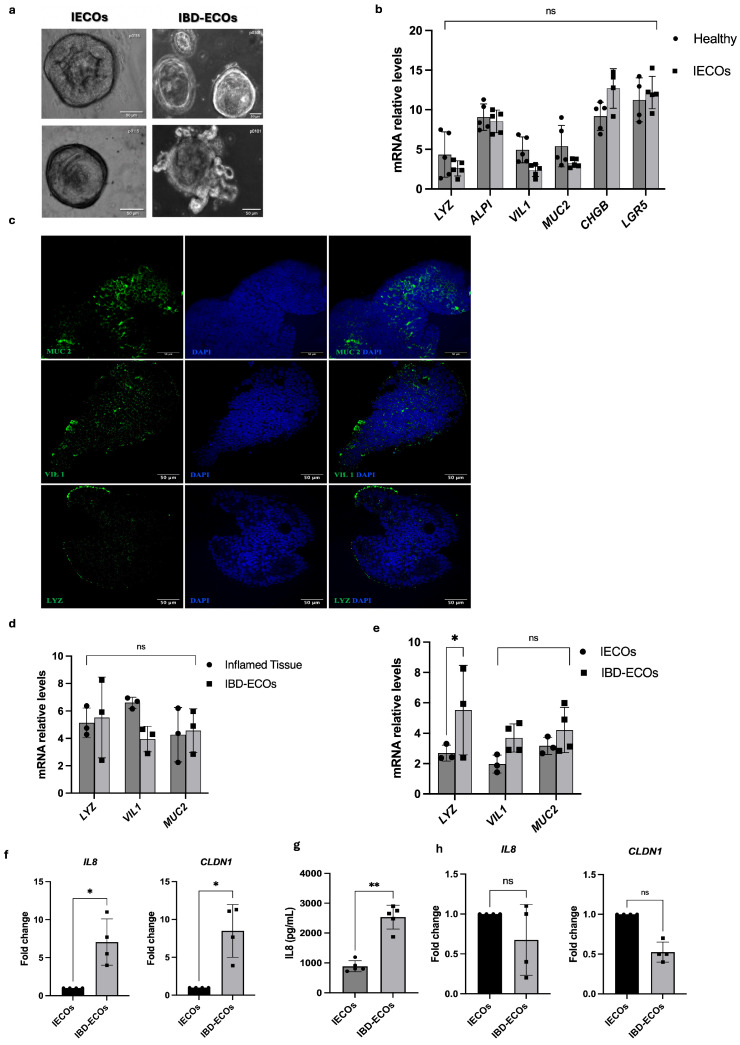
Intestinal organoids recapitulate the tissue of origin. (**a**) Representative brightfield images of intestinal organoids from non-IBD and IBD tissues of IBD patients (day 15). Scale bar: 50 μm; (**b**) qRT-PCR analysis of intestinal cytotype markers in tissue and organoids (n = 5). Bars represent mean ± SD. Dots indicate individual biological replicates; each measured as the average of technical replicates; (**c**) immunofluorescence staining in IECOs confirmed the cytotype markers protein expression. The green color confirmed the marker expression in DAPI counterstained organoids. Comparison of intestinal cytotype markers expression by qRT-PCR between IBD-ECOs vs. inflamed tissue (**d**) and non-inflamed (IECOs) vs. inflamed organoids (IBD-ECOs) (n = 3) (**e**); (**f**) Evaluation of IL8 and CLAUDIN1 (CLDN1) in IECOs and IBD-ECOs by qRT-PCR analysis; (**g**) Quantification of IL8 protein in medium of IBD-ECOs and IECOs by ELISA assay; (**h**) Assessment of inflammation-related genes in IECOs and IBD-ECOs after 4 splitting by qRT-PCR analysis. n = 3 independent patient organoid lines; Bars represent mean ± SD. Dots indicate individual biological replicates; each measured as the average of technical replicates. Statistical analysis was performed using the *t*-test. Significance levels: ** *p* < 0.01, * *p* < 0.05.

Intestinal epithelial cell organoids (IECOs) were successfully established from biopsies of both inflamed and non-inflamed intestinal segments of IBD patients (Table S1) according to well-established protocols [13,14,15]. Within 2 weeks, epithelial cell organoids derived from inflamed tissues (IBD-epithelial cell organoids, IBD-ECOs) and non-inflamed tissues (IECOs) exhibited a distinctive colon morphology with a hollow internal lumen typical of intestinal organoids (Figure 1a). Of note, organoid cultures from different donors showed different structural complexities, suggesting specific differences between patients, according to Arnauts et al [24]. To determine whether patient-derived organoids were similar to the tissue of origin in terms of cellular composition, we evaluated the expression of intestinal epithelial cell marker genes such as leucine-rich repeat-containing G-protein coupled receptor 5 (*LGR5*, intestinal stem cell), Villin 1 (*VIL1*) and Alkaline Phosphatase Intestinal (*ALPI*, enterocytes), Mucin 2 (*MUC2*, Goblet cells), Chromogranin B (*CHGB*, enteroendocrine cells), and Lysozyme (*LYZ*, Paneth cell). As shown in Figure 1b,c, IECOs faithfully reproduce key features of the epithelial cellular composition of the tissue of origin, suggesting that they also maintain the original architecture and function of their in vivo counterparts. Next, we investigated the cellular composition of IBD-ECOs, and we found, interestingly, that they retain a similar cell ratio of the inflamed tissue (Figure 1d). Moreover, we observed a statistically significant increase in Lysozyme mRNA levels when we compared IBD-ECOs with IECOs. Overall, our data indicate that IBD-ECOs maintain the cellular complexity of the tissue of origin while exhibiting a slight variation in the proportion of Paneth cells (Figure 1e). Next, we evaluated the inflammatory status of IBD-ECOs and found that, as expected, they displayed increased levels of *IL-8*, a systemic marker of inflammation and *CLDN-1*, a transmembrane protein whose increase is associated with gastrointestinal inflammation, when compared to non-IBD IECOs (Figure 1f,g), showing that organoids mimic in vitro the pathological conditions of the patients. However, as already observed in Arnauts K et al., we found that IBD-ECOs lose their inflammatory phenotype after 3–4 splitting (Figure 1h), although they retain their original cellular complexity. Overall, these data showed that patient-derived organoids resemble the in vitro patient condition and represent a suitable model to evaluate the anti-inflammatory activity of chemical drugs and natural compounds.

### 3.2. Ozoile Is a Novel Natural Product That Exerts Anti-Inflammatory and Antioxidant Activity on Intestinal Organoids

To assess the potential to use IECOs and IBD-ECOs in screening for anti-inflammatory molecules, we took advantage of the collaboration with Erbagil s.r.l. Telese Terme, Italy, which provided us with Ozoile, a compound made of a pool of stable ozonides (oxygen-rich lipid molecules) obtained through a patented green technology by reacting ozone with the olefin bonds of the fatty acids present in organic extra virgin olive oil [12]. Interestingly, Ozoile was previously shown to have anti-inflammatory, antimicrobial, and regenerative properties [25,26,27]. Having confirmed that Ozoile displays no cytotoxicity and genotoxicity in our experimental conditions (Figure S1a–d), we evaluated its anti-inflammatory activity on IECOs and, for comparison, on human colon adenocarcinoma cells (Caco-2), a widely used cellular model for studying intestinal inflammation. To this aim, we treated both 2D and 3D models with TNFα, a major pro-inflammatory cytokine that plays a central role in the pathogenesis of both Crohn’s disease and ulcerative colitis, and it represents a key factor to elicit inflammatory responses in experimental intestinal models [28].

**Figure 2:**
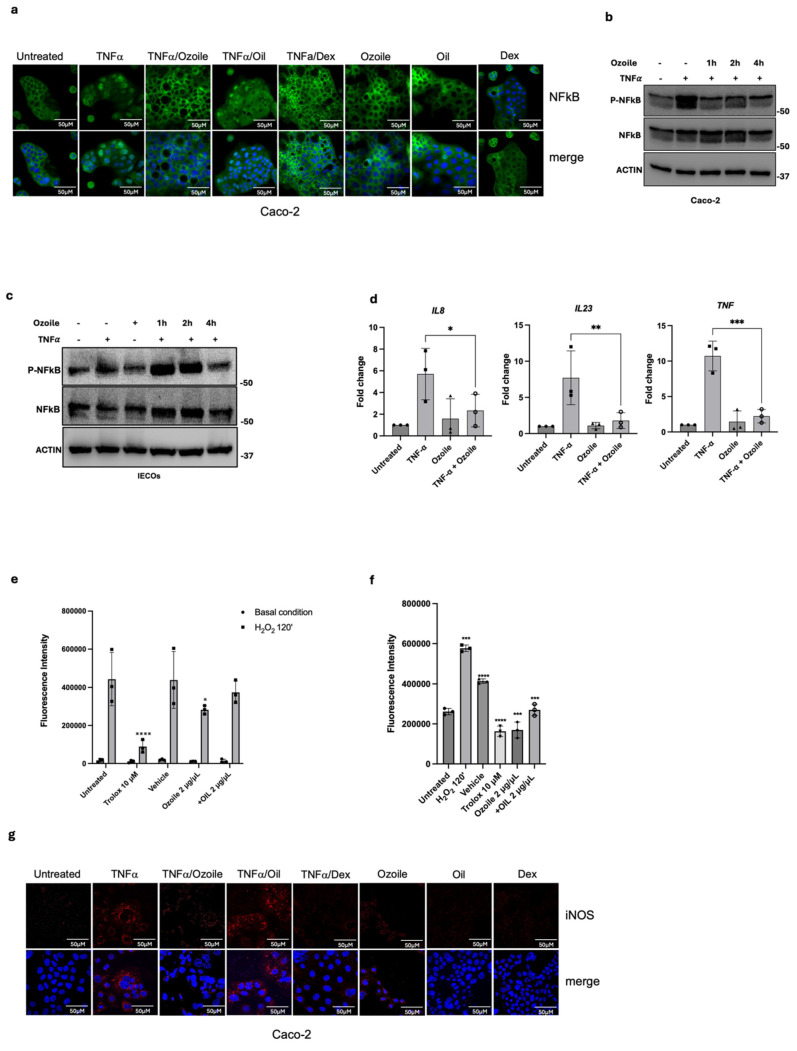
Ozoile has anti-inflammatory and antioxidant activity. (**a**) Measurement of NF-κB activation by assessing the nuclear translocation by immunofluorescence assay (green) and the phosphorylation levels upon TNFα-Ozoile treatment, at several time points, through Western blot in Caco-2 (**b**) and IECOs (**c**). (**d**) Evaluation of inflammatory cytokines, by qRT-PCR analysis in IECOs treated with TNFα alone for 4 h or in combination with Ozoile for 24 h, n = 3 independent IECO lines; Bars represent mean ± SD. Dots indicate individual biological replicates, each measured as the average of technical replicates. Antioxidant activity assessed by the DCFDA assay in Caco-2 cells treated with the indicated compounds under two experimental conditions: (**e**) pre-treatment for 4 h prior to oxidative stress induction with hydrogen peroxide (H_2_O_2_), and (**f**) co-treatment with indicated compounds and H_2_O_2_ simultaneously for 2 h. Trolox was used as a positive control, and isopropanol (vehicle), the solvent for the natural compounds as a negative control. DCFDA fluorescence intensity was measured after 45 min of incubation. Statistical analysis was performed using two-way ANOVA followed by Tukey’s multiple comparisons test. Bars represent mean ± SD. Dots indicate individual biological replicates; each measured as the average of technical replicates. Significance levels: **** *p* < 0.0001, *** *p* < 0.001, ** *p* < 0.01, * *p* < 0.05. (**g**) Expression of iNOS (red) was evaluated by immunofluorescence in Caco-2 cells treated as indicated by the red color. DAPI was used to stain nuclei (blue color).

As reported in Figure 2a,b, we observed a modulation of NF-κB phosphorylation and a loss of its nuclear translocation when Caco-2 cells were treated with Ozoile. Interestingly, we observed the same anti-inflammatory effect when Ozoile was administered to TNFα-treated IECOs, leading to a reduction in IL-8, IL-23, and TNFα mRNA levels as well as the NF-κB phosphorylation (Figure 2c,d). Furthermore, since inflammation is associated with Reactive Oxygen Species (ROS) production and activation of Nitric Oxide Synthase (NOS) enzymes [29,30], we evaluated whether Ozoile modulates ROS levels and the expression of inducible Nitric Oxide Synthase (iNOS), a direct transcriptional target of NF-κB [30]. We found that ROS levels were reduced in the presence of Ozoile, along with a pronounced decrease in iNOS expression (Figure 2e–g), suggesting that Ozoile has antioxidant activity and modulates inflammation responses upstream of NF-κB. Overall, our data demonstrate for the first time the anti-inflammatory activity of Ozoile on a 3D in vitro model of IBD and suggest that intestinal organoids can be used as a preclinical model for the screening of anti-inflammatory drugs.

### 3.3. Ozoile Has an Epithelial Barrier Modulatory Effect

**Figure 3:**
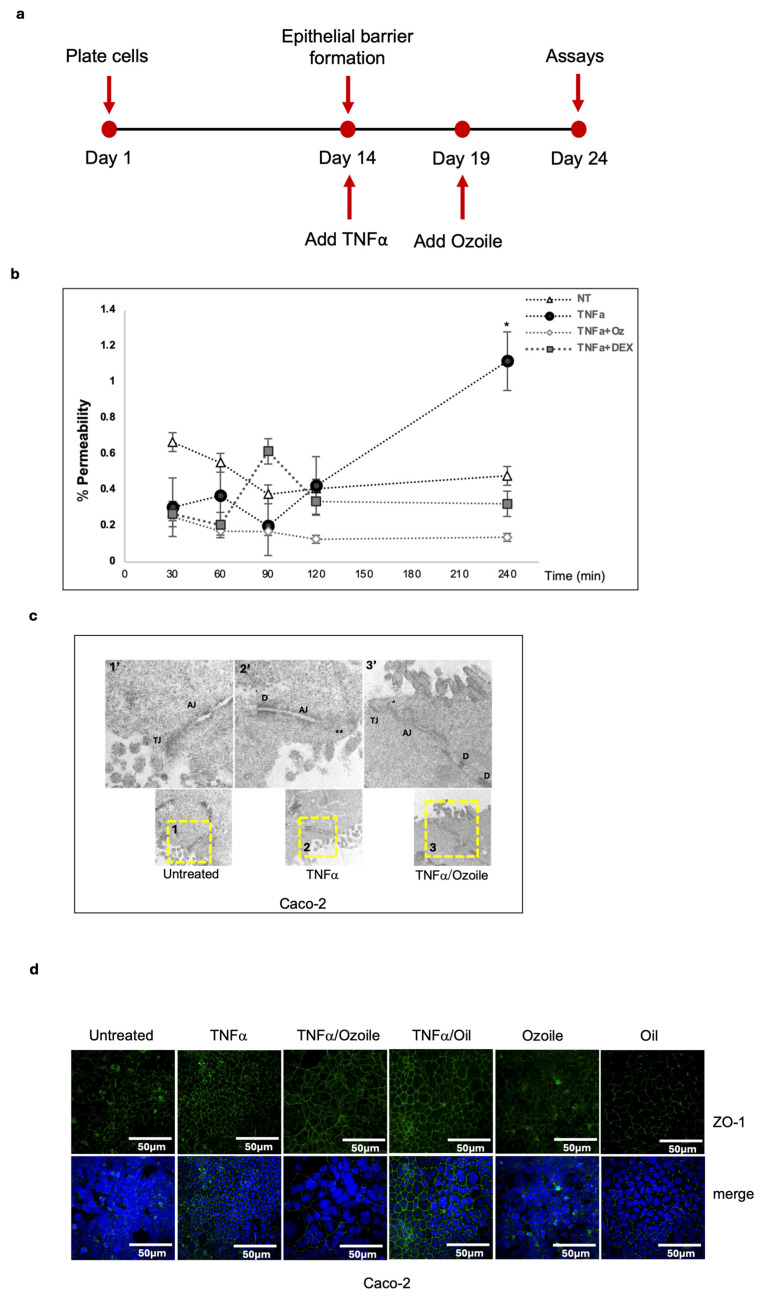
Ozoile reduces epithelial barrier permeability. (**a**) Schematic representation of the experimental design to differentiate Caco-2 cells into enterocytes; Differentiated enterocytes were treated as indicated. Apical-to-basal paracellular permeability was evaluated using Lucifer Yellow (**b**). Statistical analysis was performed using two-way ANOVA followed by Tukey’s multiple comparisons test. Significance levels: * *p* < 0.05; where not indicated, differences are not significant; (**c**) Tight junctions were evaluated by Transmission Electron Microscopy analyses of ultrathin sections. Yellow-dashed squares indicate the regions shown in the high-magnification images. TJs: Tight-Junctions; AJ: Apical Junctions; **: aberrant Tight-Junctions; D: Desmosome. (**d**) Differentiated enterocytes were treated as indicated, and tight junctions were stained by IF using the anti-ZO-1 antibody (green). Nuclei were stained with DAPI (blue).

Chronic inflammation in IBD patients is responsible for intestinal epithelial barrier permeability, commonly known as ‘leaky-gut’. The resulting porous barrier allows harmful substances to pass through, promoting mucosal inflammation. Hence, when considering a therapeutic strategy for IBD, the reduction in the inflammatory cascade alone is not sufficient if not accompanied by the concomitant restoration of epithelial barrier integrity [31,32]. To address the capability of Ozoile Food^®^ to heal epithelial barrier leakage, we differentiated Caco-2 cells into enterocytes to mimic the intestinal barrier in vitro (Figure 3a) [33]. As expected, the Caco-2 monolayer exhibited the typical cellular polarization of the epithelial barrier and microvilli formation (Figure S2). As shown in Figure 3b, TNFα-induced pro-inflammatory stimulation increased barrier permeability, which was restored following treatment with Ozoile. Accordingly, we observed an alteration of ZO-1 deposition at the tight-junctions (TJs) shown by an aberrant IF signal in inflamed cells that was probably due to a preferential apical deposition of ZO-1 in TNFα-treated cells, as confirmed by transmission electron microscopy (TEM) analysis (Figure 3c,d). Strikingly, when cells were treated in combination with Ozoile, we found that ZO-1 deposition at TJs was similar to that of control cells (Figure 3d) and observed the restoration of normal TJs, as shown in Figure 3c. In general, these data showed an unprecedented assayed activity of stable ozonides. We found that the Ozoile is capable of restoring intestinal barrier permeability and TJs, although in an in vitro assay, suggesting its potential as a natural adjunct in treating IBD symptoms.

### 3.4. A Luciferase-Based IBD-like Model for the Preliminary Screening of Anti-Inflammatory Molecules/Substances

**Figure 4:**
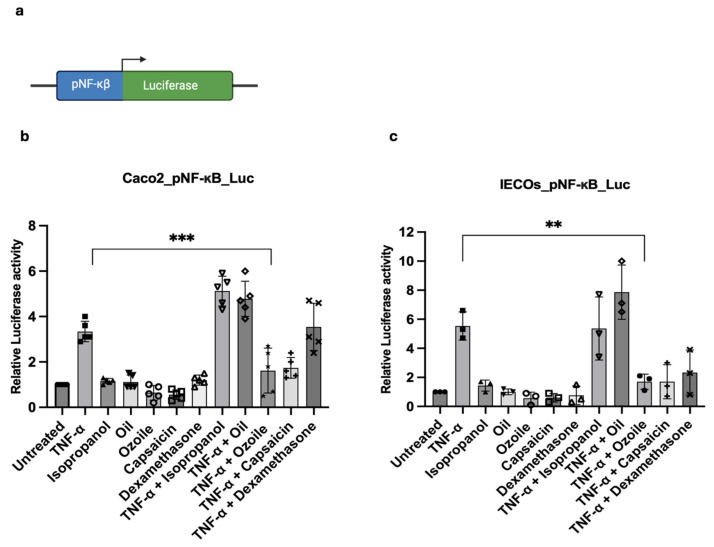
Luciferase-based IBDF-like system is suitable to evaluate substances/molecules anti-inflammatory activity. (**a**) Schematic representation of the NF-κB reporter system; Luciferase assay to assess Ozoile anti-inflammatory activity using Caco-2-NF-κB-Luc (n = 5 replicates) (**b**) and IECOs-NF-κB-Luc (n = 3 replicates) reporter models (**c**) treated with TNFα alone for 4 h and in combination with Ozoile for 24 h, and compared to the untreated. Capsaicin and dexamethasone were used as positive controls, and isopropanol, the solvent for the natural compounds, was used as a negative control. Bars represent mean ± SD. Dots indicate individual biological replicates; each measured as the average of technical replicates. Statistical analysis was performed using one-way ANOVA. Significance levels: *** *p* < 0.001, ** *p* < 0.01.

Organoids, as advanced preclinical models, represent a powerful tool to replicate human organs in vitro; nonetheless, their use in drug-screening pipelines remains limited. To contribute to an advancement in this field, we sought to develop a luciferase-based IBD-like model using human intestinal organoids for the screening of molecules with anti-inflammatory properties. To this aim, we first generated a genetically modified Caco-2 cell line using the pNF-κBLucPuro lentivirus (Figure 4a) and tested whether Caco-2-pNF-κBLuc cells were responsive to TNF-induced inflammation. As shown in Figure 4b, Caco2-pNF-κBLuc cells were responsive to the inflammatory stimuli, and, of note, Caco2-pNF-κBLuc cells were also sensitive to Ozoile. Next, we generated IECOs-pNF-κBLuc organoid lines and assessed whether they were responsive to inflammation stimuli. Thus, upon TNFα treatment, we observed an increase in luciferase activity, indicating that the model is responsive to inflammatory cytokines (Figure 4c). To address whether IECOs-pNF-κBLuc organoids could be used as a model for the screening of anti-inflammatory molecules, we treated IECOs-pNF-κBLuc organoids with Ozoile, and we further confirmed its anti-inflammatory properties. Notably, we observed a similar result upon treatment with other well-known anti-inflammatory agents, such as Dexamethasone and Capsaicin (Figure 4b,c). Particularly, dexamethasone is a synthetic glucocorticoid with potent anti-inflammatory effects as it inhibits NF-κB activation, preventing the transcription of pro-inflammatory genes and inflammatory responses in intestinal environments [34]. Capsaicin, a natural compound derived from chili peppers, has been shown to exert anti-inflammatory effects relevant to intestinal inflammation in both in vitro and in vivo studies, as it reduces levels of pro-inflammatory cytokines through the inhibition of NF-κB signaling pathways [35]. Overall, our data demonstrate that IECOs-pNFκBLuc organoids represent a suitable luciferase-based IBD-like model for the preliminary screening of anti-inflammatory drugs.

## 4. Discussion

Current treatments for inflammatory bowel disease (IBD), a multifactorial, chronic and progressive disorder comprising Ulcerative Colitis and Crohn’s Disease, rely on a range of pharmacological agents and, more recently, on various biologics. Despite the number of therapeutic strategies, definitive gold-standard therapy is still missing, and IBD management remains challenging, particularly due to the limited data on the long-term safety and adverse effects of the standard medications. These shortcomings significantly affect patients’ quality of life and, consequently, lead to an increasing burden on countries’ healthcare systems. Therefore, the identification and development of alternative therapeutic options, as well as more appropriate experimental models for therapeutic drug screening, remain critical research priorities aimed at improving clinical outcomes and patient quality of life [36]. Recent studies have highlighted the potential role of natural molecules in modulating inflammatory response and improving clinical parameters [37,38,39]. Although interest in natural molecules is steadily growing, only a limited number of studies are currently available, most of which have been conducted on immortalized cell lines, while preclinical research and clinical trials remain scarce [40,41].

In this work, we combined the use of a novel natural compound, namely Ozoile, a patented formulation made of stable ozonides combined with extravirgin olive oil, with the use of an innovative preclinical model such as patient-derived organoids, to evaluate the potential of using natural compounds as therapeutic supplements for the IBD management and to lay the grounds for the establishment of an innovative IBD-like model platform for the preliminary screenings of anti-inflammatory molecules.

We established intestinal organoids from IBD patients and demonstrated that they recapitulate in vitro key epithelial features observed in the patients. Nonetheless, we observed that after a few passages in culture, IBD epithelial cell organoids (IBD-ECOs) lose the inflammatory molecular signature, although mimicking the inflamed status upon appropriate stimuli, as in Arnauts et al. [24]. Using patient-derived intestinal organoids, we addressed for the first time the anti-inflammatory properties of Ozoile on a three-dimensional preclinical model, suggesting its potential as a candidate adjunct for further preclinical evaluation. Of note, chronic inflammation in IBD patients is responsible for an increased intestinal epithelial barrier permeability; thus, therapeutic options for IBD management need to accomplish both the reduction in inflammation and the parallel functional restoration of epithelial barrier integrity. In our study, we identified a previously uncharacterized property of Ozoile, although ozonides present in Ozoile showed local anti-inflammatory and regenerative features on inflamed skin and immortalized tumor cells [25,26]. We differentiated enterocytes in vitro and demonstrated that Ozoile modulates epithelial barrier function, as indicated by reduced paracellular permeability and modulation of tight junction-associated markers, suggesting employment of Ozoile as a potential adjuvant strategy for IBD treatment. Furthermore, we advanced organoid research by establishing a luciferase-based IBD-like organoid model platform for preliminary screening of anti-inflammatory drugs, as organoids represent a promising tool in preclinical research and serve as a tool for screening both natural and synthetic compounds. We showed that IBD-like Luc-organoids recapitulate key features of TNFα-induced inflammatory signaling and properly respond to anti-inflammatory molecules, suggesting their utility as a suitable system for preliminary drug evaluation, as they enabled precise and dynamic monitoring of therapeutic responses. From a mechanistic perspective, TNFα stimulation of intestinal epithelial models is well established to activate canonical inflammatory signaling pathways, most prominently NF-κB, as well as downstream MAPK cascades, which together regulate epithelial inflammatory and stress-associated transcriptional programs [42,43]. In parallel, TNFα has been shown to impair epithelial barrier integrity through defined signaling mechanisms that affect junctional organization and permeability [32,44]. Within this established framework, the effects observed upon Ozoile co-treatment can be conservatively interpreted as modulation of the epithelial response to TNFα, potentially influencing the magnitude or downstream consequences of these pathways. Nonetheless, the observed reduction in NF-κB activation, as indicated by decreased phosphorylation and increased cytoplasmic retention upon Ozoile co-treatment, is consistent with modulation of TNFα-dependent signaling events occurring upstream or at the level of NF-κB activation. Based on this evidence, we cannot exclude the possibility that Ozoile may interfere with upstream TNFα-dependent signaling mechanisms; however, this hypothesis requires direct experimental validation [45,46,47]. Importantly, these considerations are hypothesis-generating, as the present study was designed as a functional screening platform and does not directly interrogate pathway activation. Building on these findings, we explored the application of engineered patient organoids as a preliminary epithelial screening platform for anti-inflammatory molecules, as well as the Ozoile effect in relevant experimental systems. Given its efficacy in reducing inflammatory cytokines, its properties in restoring epithelial barrier permeability, and its favorable response profiles in patient-derived models, further investigation through preclinical and clinical studies is warranted [11,48,49]. Notably, we are aware that this study has several limitations that should be acknowledged. The sample size of patients enrolled in this study is limited. Furthermore, the organoid system used here is composed exclusively of intestinal epithelial cells and therefore lacks immune cells, stromal components, microbiota, and systemic cues that contribute to intestinal inflammation in vivo. In addition, the control biopsies used to generate organoids were obtained from endoscopically and histologically non-inflamed regions of IBD patients rather than from truly healthy non-IBD individuals. Although this approach allows the generation of patient-matched epithelial models, it may not fully represent physiologically normal intestinal mucosa, and future studies including organoids derived from non-IBD tissues will be important to further validate these observations. In addition, in our study, inflammatory responses were induced using TNFα as a single cytokine stimulus, representing a simplified and controlled system inflammatory trigger that does not recapitulate the full complexity of IBD, as it involves multiple cytokines. Nevertheless, TNFα is widely used in epithelial and organoid-based models as a controlled and reproducible trigger of inflammatory stress. Future extensions of this platform could incorporate cytokine combinations and immune–epithelial co-culture systems to better capture disease complexity. In addition, while organoids from independent donors were included, expanding the number of patient-derived lines will be essential to apply organoids as a robust screening tool and to capture inter-individual variability. Moreover, since capsaicin and dexamethasone are only partially representative of current clinical practice, additional control experiments employing other biologic agents or JAK inhibitors would be necessary to strengthen the findings regarding the potential therapeutic option of Ozoile (either primary or adjunctive) for inflammatory bowel disease (IBD).

Furthermore, the effects of Ozoile were assessed exclusively in vitro, and no in vivo validation was performed. While this reductionist approach supports the development of a controlled epithelial screening platform, further studies using more complex experimental models and, ultimately, clinical studies, will be required to validate the translational relevance of these findings.

In conclusion, this study highlights the relevance of organoid-based platforms as preclinical tools for mechanistic investigation and compound screening.

## Data Availability

The original contributions presented in this study are included in the article/Supplementary Materials. Further inquiries can be directed to the corresponding author.

## Supplementary Materials

The following supporting information can be downloaded at: https://www.mdpi.com/article/10.3390/antiox15060664/s1.
