# Supplementary material for: Integrated Epithelial Models Reveal Anti-Inflammatory and Barrier Modulatory Properties of Ozoile in Inflammatory Bowel Disease
Source: Antioxidants (Basel). 2026 May 25;15(6):664. doi: 10.3390/antiox15060664 (PMC13295276; doi:10.3390/antiox15060664)
Supplement: Supplementary file 1 [file antioxidants-15-00664-s001.zip › Supplementary Information.pdf]

Supplementary

Integrated epithelial models reveal anti-inflammatory and barrier modulatory properties of Ozoile in inflammatory bowel disease

Supplementary Figure S1 Preclinical Safety Evaluation of Ozoile Caco-2 cells (A) were treated with increasing concentrations of Ozoile (0.25–5 mg/ml) for 0, 24, 48, and 72h, and viability was assessed by MTT assay. B) Caco-2 cells were treated as indicated and analyzed by immunofluorescence using a-gH2AX (red signal). Nuclei were stained with DAPI. Representative images are shown. C-D) IECOs were treated with increasing concentrations of Ozoile (0.25–5 mg/ml) for 0, 24, 48, and 72h, and viability was assessed by MTT assay and microscopy imaging. Data are shown as mean ± SD (n=3). Statistical analysis was performed using a two-way ANOVA (ns = $p > 0.05$, **** = $p < 0.0001$).

Supplementary Figure S2 Caco-2 cells differentiation into enterocytes.

Caco-2 cells were treated according to the experimental design in Figure 3A. Pictures were taken at Transmission Electron Microscopy at the indicated magnifications. n: nucleus; AV: Apical Villi Representative images are shown.
