# Supplementary figures and images for: Integrated Epithelial Models Reveal Anti-Inflammatory and Barrier Modulatory Properties of Ozoile in Inflammatory Bowel Disease
Source: Antioxidants (Basel). 2026 May 25;15(6):664. doi: 10.3390/antiox15060664 (PMC13295276; doi:10.3390/antiox15060664)

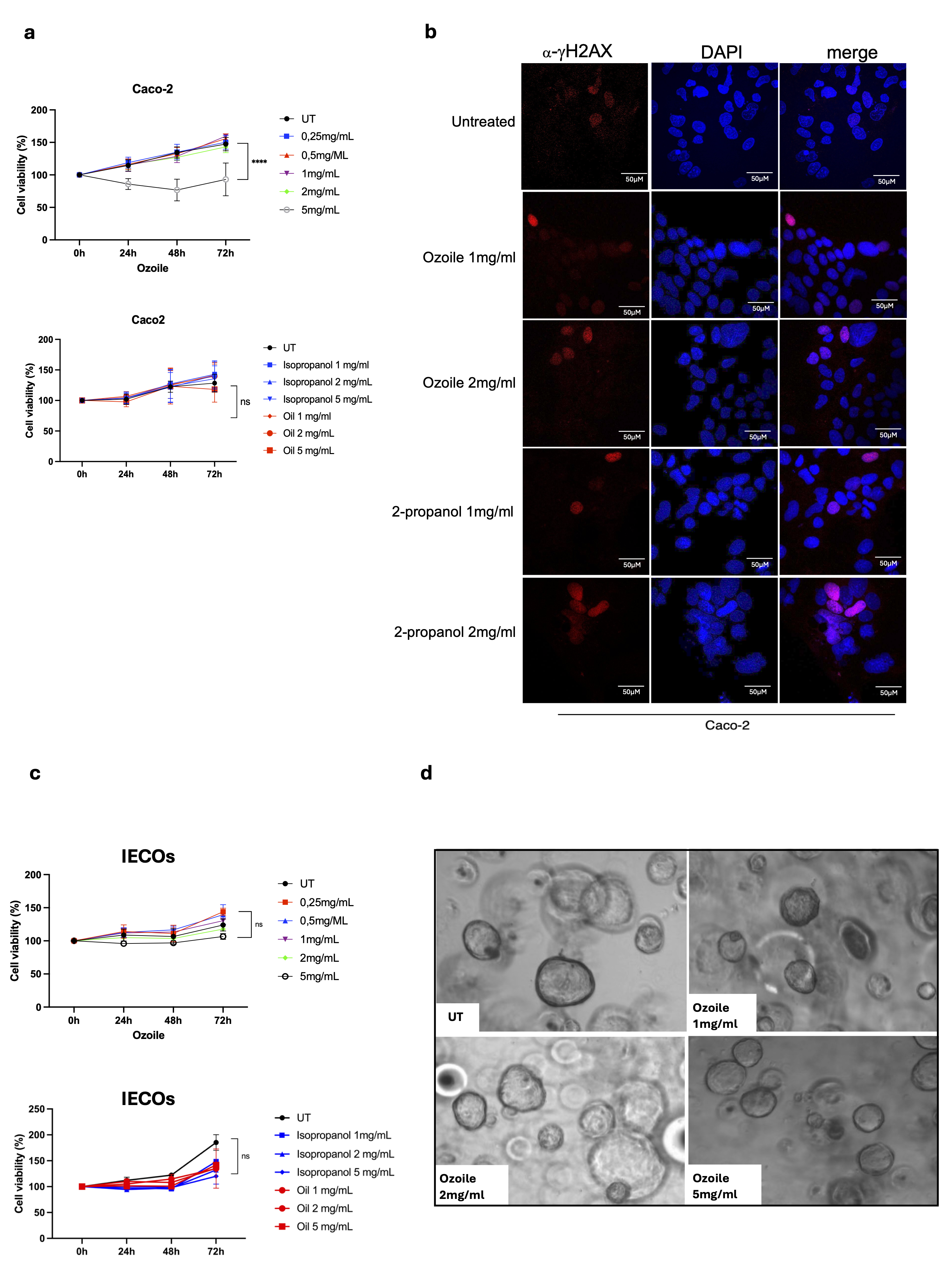

Supplement: Supplementary file 1 [file antioxidants-15-00664-s001.zip › Figure S1.tiff]

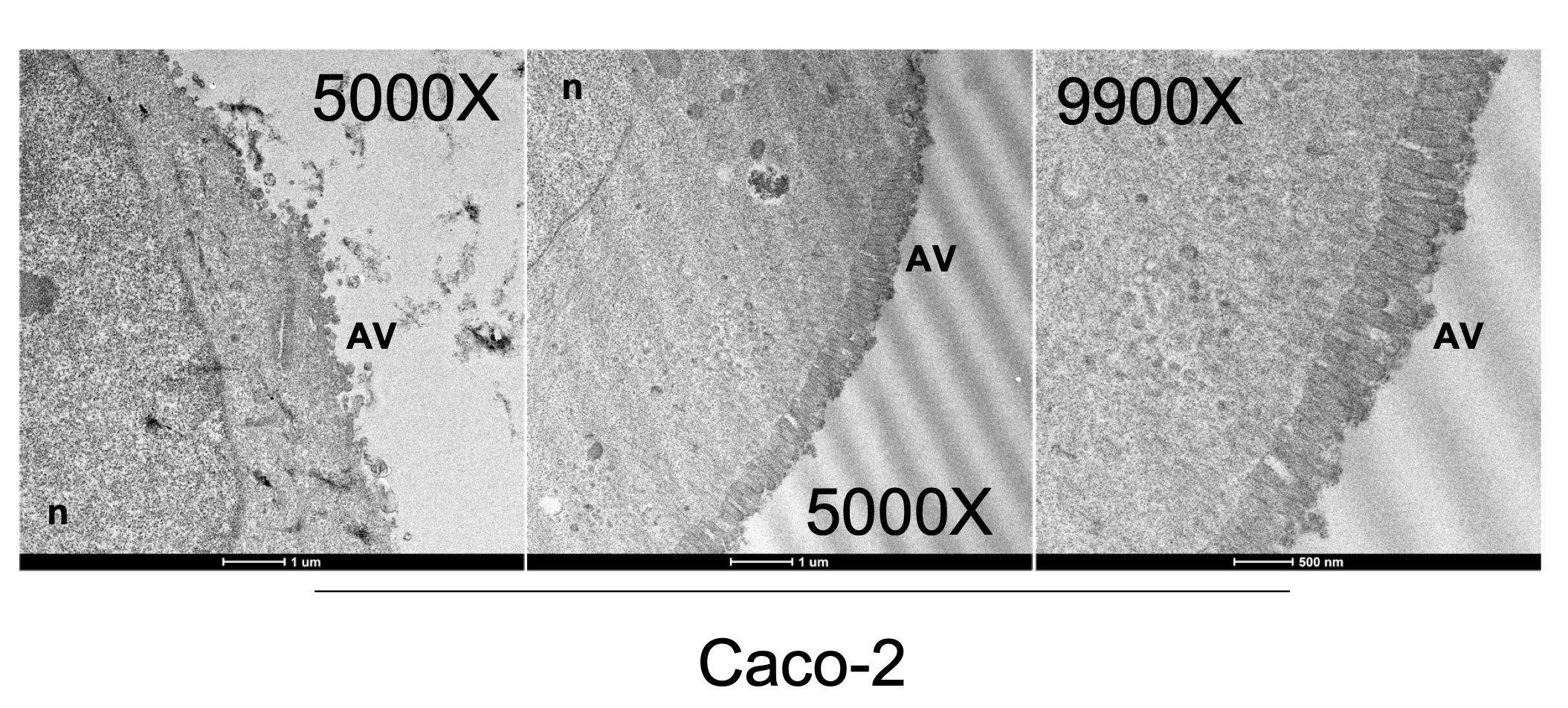

Supplement: Supplementary file 1 [file antioxidants-15-00664-s001.zip › Figure S2.tiff]
